# Supplementary material for: Safety of endovascular therapy in ischemic stroke patients ⩾90 years: A cohort study from the EVA-TRISP collaboration
Source: Eur Stroke J. 2025 Aug 13:23969873251360607. Online ahead of print. doi: 10.1177/23969873251360607 (PMC12350292; doi:10.1177/23969873251360607)
Supplement: sj-docx-1-eso-10.1177_23969873251360607 – Supplemental material for Safety of endovascular therapy in ischemic stroke patients ≽90 years: A cohort study from the EVA-TRISP collaboration [file sj-docx-1-eso-10.1177_23969873251360607.docx]

**Supplement**

**Supplementary Table 1.** Centres and time of data acquisition.

| **Centres (city, country)** | **Time table** | **Number of Patients** |
| --- | --- | --- |
| Amsterdam, Netherlands | 01/16-03/22 | 952 |
| Basel, Switzerland | 01/15-07/22 | 785 |
| Belgrad, Serbia | 01/18-12/21 | 239 |
| Berlin, Germany | 10/15-12/21 | 812 |
| Bern, Switzerland | 02/15-10/22 | 2180 |
| Bologna, Italy | 09/18-09/22 | 557 |
| Brescia, Italy | 01/15-07/21 | 503 |
| Göteborg, Sweden | 12/13-12/20 | 1387 |
| Helsinki, Finland | 01/16-12/20 | 918 |
| Jerusalem, Israel | 01/15-03/22 | 427 |
| Lausanne, Switzerland | 01/15-12/22 | 976 |
| Lisbon, Portugal | 01/16-04/22 | 575 |
| Lugano, Switzerland | 02/15-01/23 | 452 |
| Modena, Italy | 01/15-12/20 | 680 |
| St. Gallen, Switzerland | 01/15-01/22 | 853 |
| Zürich, Switzerland | 01/15-12/22 | 1010 |
| **Total** |  | 13’303 |

**Supplementary Table 2.** Details of A) site of vessel occlusion and B) EVT complications.

| **Variable** | | **All (n=13306)** | **Patients <90 years (n=12411)** | **Patients ≥90 years (n=892)** | **Raw p-value** | **Adj. p-value** | **Missing Data** |
| --- | --- | --- | --- | --- | --- | --- | --- |
| **A)** | Internal carotid artery | 2427/11375 (21.3%) | 2258/10591 (21.3%) | 169/784 (21.6%) | 0.912 | 1 | 1823 (14.7%) / 108 (12.1%) |
|  | Medial cerebral artery M1 | 5425/11375 (47.7%) | 5011/10591 (47.3%) | 414/784 (52.8%) | 0.003 | 0.027 |  |
|  | Medial cerebral artery M2 | 3008/11375 (26.4%) | 2794/10591 (26.4%) | 214/784 (27.3%) | 0.604 | 1 |  |
|  | Anterior cerebral artery | 214/11375 (1.9%) | 197/10591 (1.9%) | 17/784 (2.2%) | 0.633 | 1 |  |
|  | Posterior cerebral artery | 399/11375 (3.5%) | 380/10591 (3.6%) | 19/784 (2.4%) | 0.108 | 0.86 |  |
|  | Basilar artery | 748/11375 (6.6%) | 719/10591 (6.8%) | 29/784 (3.7%) | <0.001 | 0.008 |  |
|  | Vertebral artery | 260/11375 (2.3%) | 255/10591 (2.4%) | 5/784 (0.6%) | 0.002 | 0.017 |  |
|  | Other localization | 761/11375 (6.7%) | 709/10591 (6.7%) | 52/784 (6.6%) | 1 | 1 |  |
| **B)** | *All EVT complications* | *1562/9134 (17.1%)* | *1435/8517 (16.9%)* | *127/617 (20.6%)* |  |  | *3897 (31.4%) / 275 (30.8%)* |
|  | Vessel Perforation | 230/9134 (2.5%) | 213/8517 (2.5%) | 17/617 (2.8%) | 0.798 | 1 |  |
|  | Vasospasm | 299/9134 (3.3%) | 289/8517 (3.4%) | 10/617 (1.6%) | 0.023 | 0.208 |  |
|  | Dissection | 193/9134 (2.1%) | 178/8517 (2.1%) | 15/617 (2.4%) | 0.672 | 1 |  |
|  | Haemorrhage (SAB or ICH) | 250/9134 (2.7%) | 221/8517 (2.6%) | 29/617 (4.7%) | 0.003 | 0.027 |  |
|  | Device detachment or misplacement | 17/9134 (0.2%) | 17/8517 (0.2%) | 0/617 (0%) | 0.531 | 1 |  |
|  | Embolization to new territory | 431/9134 (4.7%) | 392/8517 (4.6%) | 39/617 (6.3%) | 0.065 | 0.585 |  |
|  | Access-site complications | 210/9134 (2.3%) | 186/8517 (2.2%) | 24/617 (3.9%) | 0.01 | 0.086 |  |
|  | Early reocclusion | 154/9134 (1.7%) | 145/8517 (1.7%) | 9/617 (1.5%) | 0.77 | 1 |  |
|  | Other | 146/9134 (1.6%) | 126/8517 (1.5%) | 20/617 (3.2%) | 0.001 | 0.012 |  |

**Legend:** SAB = Subarachnoidal bleeding, ICH = intracerebral hemorrhage. The χ2 test was used to compare the age groups. Adjusted p-values are derived from Bonferronis correction applied across the stroke localization and the EVT complications separately (and excluding the row showing all EVT complications). Missing data are given per group (young versus old) separately. Most common “Other” EVT complications were presence of multiple complications or not further specified complications followed by cardiac problems, change in type of anesthesia and profound access-site complications.

| **Supplementary Table 3.** Full models of the main analysis with all variables. Odds ratios and 95%-confidence interval boundaries are rounded to 2 digits. If the rounded value is 1, we denote values smaller than 1 with (<1) and values larger than 1 with (1>). | | | | |
| --- | --- | --- | --- | --- |
| **Variable** | **Odds Ratio** | **Lower 95%-CI** | **Upper 95%-CI** | **p-value** |
| **Poor outcome at 3 months in survivors** | | | | |
| (Intercept) | 0.1 | 0.08 | 0.13 | <0.001 |
| Age (old vs young) | 2.35 | 1.87 | 2.97 | <0.001 |
| NIHSS at Admission | 1.09 | 1.09 | 1.1 | <0.001 |
| Sex (male vs female) | 0.86 | 0.78 | 0.94 | <0.001 |
| EVT Complications (Yes) | 1.28 | 1.1 | 1.49 | 0.001 |
| Unsuccessful Recanalization (Yes) | 2.13 | 1.9 | 2.4 | <0.001 |
| Prestroke mRS | 1.04 | 0.99 | 1.09 | 0.094 |
| Bridging (Yes) | 0.67 | 0.61 | 0.73 | <0.001 |
| Admission Glucose | 1.07 | 1.05 | 1.09 | <0.001 |
| Atrial Fibrillation (Yes) | 1.19 | 1.08 | 1.31 | <0.001 |
| Prior Ischemic Stroke (Yes) | 1.19 | 1.04 | 1.36 | 0.013 |
| Time to Groin (per 30 minutes) | 1(>1) | 1(>1) | 1.01 | 0.003 |
| **Death at 3 month** | | | | |
| (Intercept) | 0.03 | 0.02 | 0.03 | <0.001 |
| Age (old vs young) | 3.04 | 2.6 | 3.55 | <0.001 |
| NIHSS at Admission | 1.09 | 1.08 | 1.1 | <0.001 |
| Sex (male vs female) | 1.08 | 0.99 | 1.19 | 0.083 |
| EVT Complications (Yes) | 1.73 | 1.51 | 1.99 | <0.001 |
| Unsuccessful Recanalization (Yes) | 1.67 | 1.5 | 1.86 | <0.001 |
| Prestroke mRS | 1.44 | 1.39 | 1.5 | <0.001 |
| Bridging (Yes) | 0.64 | 0.58 | 0.7 | <0.001 |
| Admission Glucose | 1.07 | 1.05 | 1.09 | <0.001 |
| Atrial Fibrillation (Yes) | 1.08 | 0.98 | 1.18 | 0.127 |
| Prior Ischemic Stroke (Yes) | 1.12 | 0.99 | 1.28 | 0.071 |
| Time to Groin (per 30 minutes) | 1(>1) | 1(>1) | 1.01 | 0.142 |
| **Symptomatic intracranial hemorrhage** | | | | |
| (Intercept) | 0.02 | 0.01 | 0.03 | <0.001 |
| Age (old vs young) | 0.92 | 0.66 | 1.28 | 0.618 |
| Sex (male vs female) | 0.99 | 0.83 | 1.17 | 0.882 |
| NIHSS at Admission | 1.03 | 1.01 | 1.04 | <0.001 |
| EVT Complications (Yes) | 3.47 | 2.84 | 4.23 | <0.001 |
| Bridging (Yes) | 1.15 | 0.97 | 1.36 | 0.117 |
| Admission Systolic Blood pressure (per 10 mmHg) | 1.02 | 1(>1) | 1.05 | 0.075 |
| Time to Groin (per 30 minutes) | 0.99 | 0.99 | 1(>1) | 0.148 |
| **Unsuccessful Recanalization** | | | | |
| (Intercept) | 0.54 | 0.43 | 0.67 | <0.001 |
| Age (old vs young) | 1.34 | 1.14 | 1.57 | <0.001 |
| Sex (male vs female) | 1 (<1) | 0.92 | 1.09 | 0.972 |
| NIHSS at Admission | 1.01 | 1.01 | 1.02 | <0.001 |
| EVT General anesthesia (Yes) | 1.09 | 1 (<1) | 1.19 | 0.06 |
| Bridging (Yes) | 0.99 | 0.9 | 1.08 | 0.774 |
| Admission Systolic Blood pressure (per 10 mmHg) | 0.96 | 0.95 | 0.97 | <0.001 |
| Atrial Fibrillation (Yes) | 0.86 | 0.79 | 0.95 | 0.002 |
| Diabetes Mellitus (Yes) | 0.92 | 0.82 | 1.03 | 0.147 |
| Time to Groin (per 30 minutes) | 1(>1) | 1(>1) | 1.01 | 0.009 |

| \| **Supplementary Table 4.** Subgroup analyses only for patients ≥ 90 years, full models, imputed datasets and additional binary covariate as indicated (prestroke dependency, posterior versus anterior circulation, cardioembolic versus large artery atherosclerosis). \| \| \| \| \| \| --- \| --- \| --- \| --- \| --- \| \| **Outcome** \| **Odds Ratio** \| **Lower 95%-CI** \| **Upper 95%-CI** \| **p-value** \| \|  \|  \|  \|  \|  \| \| **≥ 90 years, prestroke dependency (prestroke mRS 3-5 versus 0-2)** \| \| \| \| \| \| Poor outcome at 3 months in survivors (Yes), n = 411 \| 2.16 \| 1.36 \| 3.43 \| 0.001 \| \| Death at 3 months (Yes), n = 892 \| 0.77 \| 0.57 \| 1.05 \| 0.097 \| \| Symptomatic intracranial hemorrhage (Yes), n = 892 \| 1.44 \| 0.70 \| 2.97 \| 0.327 \| \| Unsuccessful Recanalization (Yes), n = 892 \| 0.84 \| 0.60 \| 1.16 \| 0.280 \| \|  \|  \|  \|  \|  \| \| **≥ 90 years, posterior vs anterior circulation** \| \| \| \| \| \| Poor outcome at 3 months in survivors (Yes), n = 319 \| 1.31 \| 0.39 \| 4.41 \| 0.667 \| \| Death at 3 months (Yes), n = 687 \| 1.08 \| 0.49 \| 2.39 \| 0.845 \| \| Symptomatic intracranial hemorrhage (Yes), n = 687 \| 0.64 \| 0.07 \| 5.73 \| 0.689 \| \| Unsuccessful Recanalization (Yes), n = 687 \| 1.36 \| 0.61 \| 3.06 \| 0.451 \| \|  \|  \|  \|  \|  \| \| **≥ 90 years,** **cardioembolic vs large artery atherosclerosis** \| \| \| \| \| \| Poor outcome at 3 months in survivors (Yes), n = 314 \| 0.43 \| 0.16 \| 1.12 \| 0.085 \| \| Death at 3 months (Yes), n = 648 \| 0.82 \| 0.45 \| 1.46 \| 0.493 \| \| Symptomatic intracranial hemorrhage (Yes), n = 648 \| 0.59 \| 0.24 \| 1.44 \| 0.247 \| \| Unsuccessful Recanalization (Yes), n = 648 \| 1.16 \| 0.61 \| 2.21 \| 0.640 \|   **Supplementary Figure 1.** mRS distribution at baseline and after 3 months in patients ≥ 90 years and patients < 90 years. |
| --- | --- | --- | --- | --- | --- | --- | --- | --- | --- | --- | --- | --- | --- | --- | --- | --- | --- | --- | --- | --- | --- | --- | --- | --- | --- | --- | --- | --- | --- | --- | --- | --- | --- | --- | --- | --- | --- | --- | --- | --- | --- | --- | --- | --- | --- | --- | --- | --- | --- | --- | --- | --- | --- | --- | --- | --- | --- | --- | --- | --- | --- | --- | --- | --- | --- | --- | --- | --- | --- | --- | --- | --- | --- | --- | --- | --- | --- | --- | --- | --- | --- | --- | --- | --- | --- | --- | --- | --- | --- | --- | --- | --- | --- | --- | --- | --- | --- | --- | --- | --- |


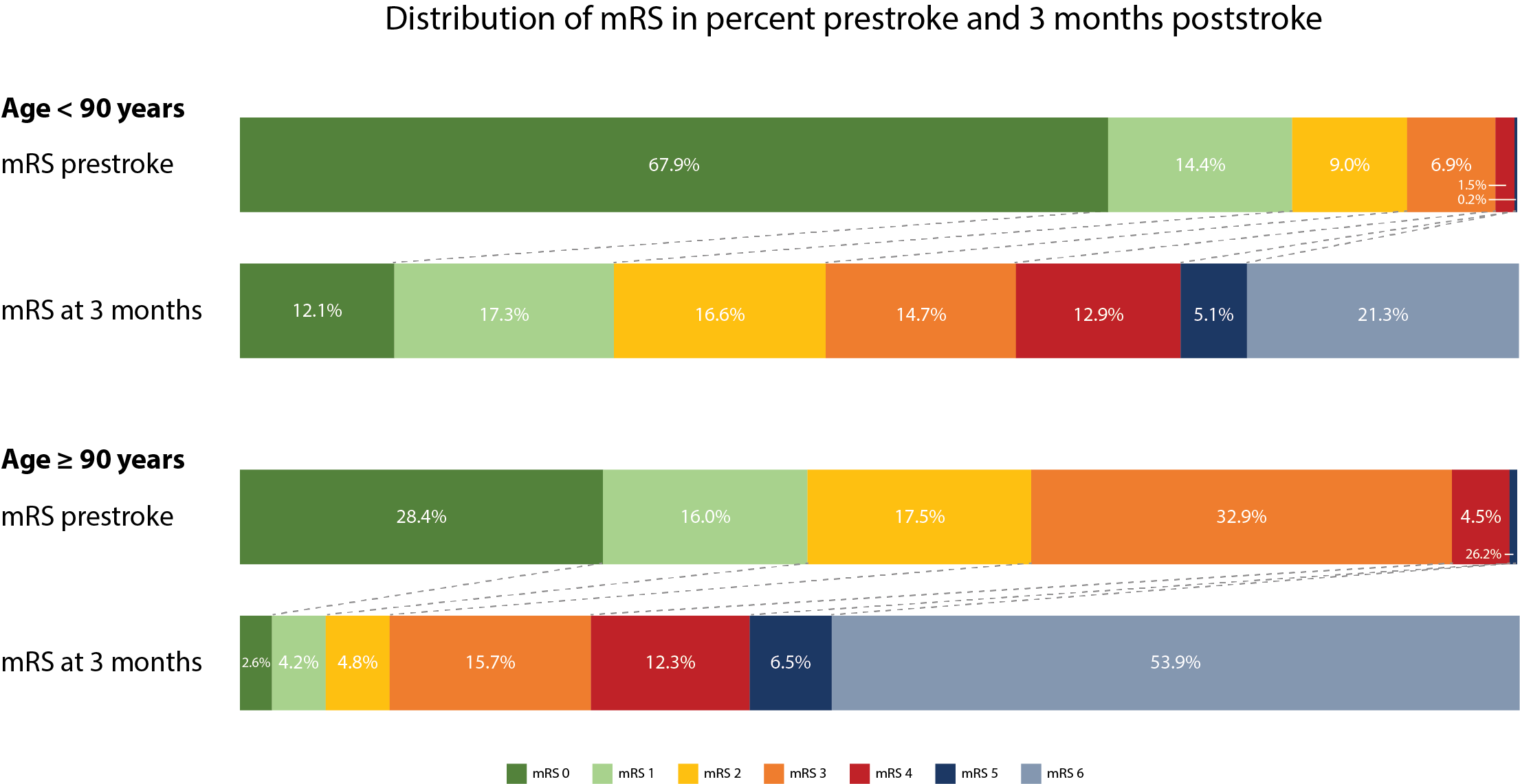


|  |
| --- |
|  |

| **Supplementary Figure 2.** Results of main analysis and sensitivity analyses. Purple: complete case analysis, cyan: imputed datasets (MICE imputation) using simple models, green: imputed datasets using the full models, orange: imputed datasets using the inversed propensity score matching models. |
| --- |

| 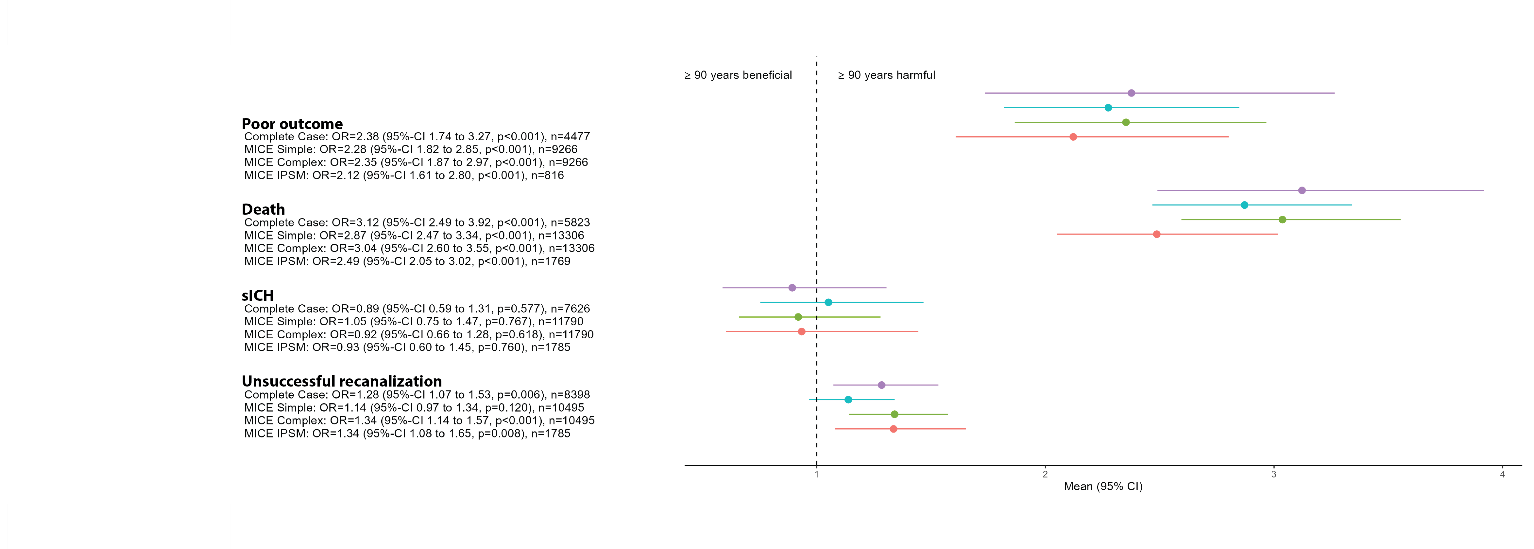 |
| --- |

**Supplementary Figure 3.** Balancing of covariates for propensity score matching before and after adjustment. A. Poor outcome in survivors. B. Death at 3 months. C. Intracranial hemorrhage. D. Unsuccessful recanalization.

| 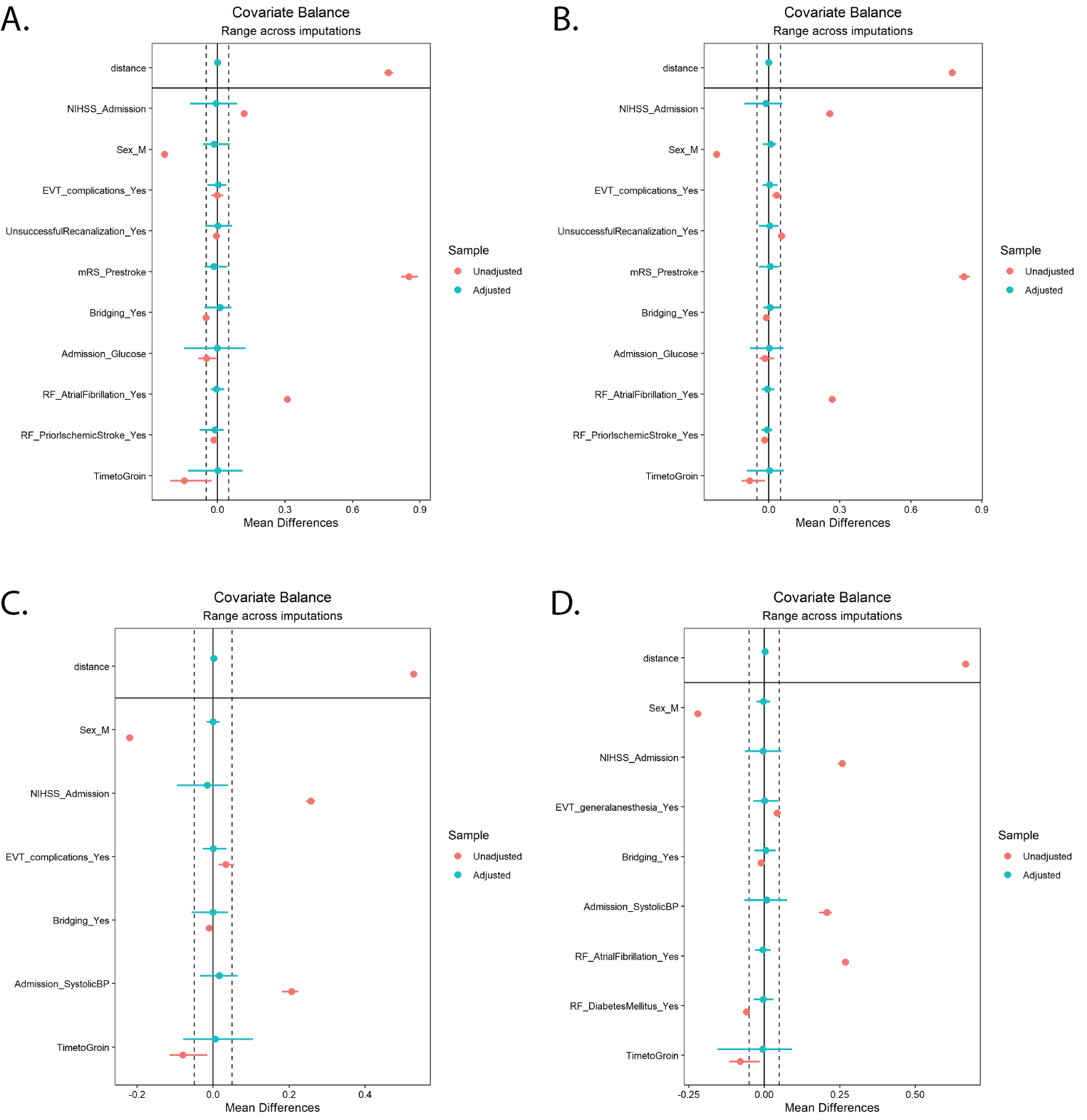 |
| --- |
